# A Systematic Review of Cognitive Function in First-Episode Psychosis, Including a Discussion on Childhood Trauma, Stress, and Inflammation

**DOI:** 10.3389/fpsyt.2013.00182

**Published:** 2014-01-08

**Authors:** Monica Aas, Paola Dazzan, Valeria Mondelli, Ingrid Melle, Robin M. Murray, Carmine M. Pariante

**Affiliations:** ^1^Division of Mental Health and Addiction, Institute of Clinical Medicine, University of Oslo, Oslo, Norway; ^2^NORMENT, K.G. Jebsen Psychosis Research Unit, Division of Mental Health and Addiction, Oslo University Hospital, Oslo, Norway; ^3^Department of Psychosis Studies, Institute of Psychiatry, King’s College London, London, UK; ^4^NIHR Biomedical Research Centre for Mental Health, South London and Maudsley NHS Foundation Trust and Institute of Psychiatry, King’s College London, London, UK; ^5^Department of Psychological Medicine, Institute of Psychiatry, King’s College London, London, UK

**Keywords:** cognition, first-episode psychosis, stress, physiological, genes × environment interactions, review of literature

## Abstract

**Objective:** To carry out a systematic review of the literature addressing cognitive functions in first-episode psychosis (FEP), divided into domains. Although this is not a full “cognitive-genetics-in-schizophrenia review,” we will also include putative ideas of mechanism(s) behind these impairments, focusing on how early stress, and genetic vulnerability may moderate cognitive function in psychosis.

**Method:** Relevant studies were identified via computer literature searches for research published up to and including January 2013, only case-control studies were included for the neurocognitive meta-analysis.

**Results:** Patients with FEP present global cognitive impairment compared to healthy controls. The largest effect size was observed for verbal memory (Cohen’s *d* effect size = 2.10), followed by executive function (effect size = 1.86), and general IQ (effect size = 1.71). However, effect sizes varied between studies.

**Conclusion:** Cognitive impairment across domains, up to severe level based on Cohen’s effect size, is present already in FEP studies. However, differences in levels of impairment are observed between studies, as well as within domains, indicating that further consolidation of cognitive impairment over the course of illness may be present. Cognitive abnormalities may be linked to a neurodevelopmental model including increased sensitivity to the negative effect of stress, as well as genetic vulnerability. More research on this field is needed.

## Introduction

With an increased awareness of the extent of cognitive impairments present in schizophrenia, cognitive dysfunction is now viewed by many as a core abnormality of the disorder (1, 2). The majority of patients with schizophrenia function at a cognitive level of at least one standard deviation below that of healthy comparison groups (3, 4). Together with a global impairment in cognitive function, specific domains show greater dysfunction, such as episodic memory, working memory, and executive function (5, 6). There are also patients with schizophrenia with cognitive scores in the normal or above-normal range, varying in different studies from 15 to 45% (6–10). Nevertheless, in the high cognitive function subgroup, 64% still have abnormal scores on at least one cognitive domain, compared to 35% of healthy controls (7). Here a profile is considered abnormal if at least two functions are more than two SDs below the normative mean, or if only a single function is extremely impaired [i.e., >3 SDs below the normative mean (6)]. Therefore, most of the high functional group demonstrates some kind of cognitive deficit compared to healthy controls.

Investigating cognitive performance in patients at the early stages of the illness has the advantage of identifying cognitive deficits more likely to reflect the neurodysfunction that underlies schizophrenia rather than possible illness or treatment related processes following the chronic course of the illness. Of course, it is still possible that prodromal symptoms, or even very short periods of psychotic symptoms, lead to cognitive changes, but investigating patients at illness onset can give new insight into the disorder, when compared to the large amount of research that has been conducted in long-term ill patients. Moreover, these studies can also help elucidate whether or not some of the cognitive changes are a consequence of long-term pharmacological treatment. In considering studies in patients with first-episode psychosis (FEP), it is important to note that some have included not just patients with schizophrenia, but also those with other psychoses. This is relevant since during this early phase diagnosis may change (11), and not including such cases would mean losing potential schizophrenia cases. It can also elicit different cognitive profiles based on the heterogeneity of the first-episode group. For example, affective psychosis may have better cognitive profile than a non-affective schizophrenia (12).

Two recent articles have reviewed cognitive function during the course of schizophrenia and other psychosis (prodromal, first-episode, and chronic illness) (13, 14). However these have a very broad scope. Instead, we decided to carry out a systematic review of cognitive function in FEP; by focusing our review we were able to go into greater depth to elicit patterns of sub area of impairment measured by effect sizes. Compared to the review article by Mesholam-Gately et al. (15), effect sizes were calculated based on controls performance from the same catchment area as the patients, thus avoiding differences in possible sample group biases independent of psychosis. This is the first review article to present in detail effect sizes comparing (subtypes) of FEP and controls on cognitive performance, divided into domains. We will also include putative mechanisms behind these impairments, and both environmental and genetic factors will be discussed.

Only case-control studies were included for the neurocognitive meta-analysis.

Further, we have included all types of FEP, not only schizophrenia (15), giving the possibility to compare “first-episode schizophrenia” versus “other psychosis” on cognitive profile at start of their first-episode of psychosis. We will also investigate if underlying cognitive factors are driving the overall cognitive performance. Finally, we will discuss possible etiological aspects, specifically genetic and environmental factors, and how they interact.

Thus, the aim of this literature review is to present in detail effect sizes comparing FEP and controls from the same catchment area on cognitive performance divided into cognitive domains; 1) investigate and discuss if specific underlying cognitive factors are driving the overall cognitive function, as well as links to disease progression; 2) discuss putative mechanisms behind our findings including sensitivity to stress, and gene environmental interactions.

## Methods

### Cognitive function in first-episode psychosis

A systematic review of the literature on cognition in FEP was conducted. Medline (PubMed) and PsycINFO bibliographic databases were used to search for articles reviewed. The search strategies involved the following keywords: “cognition and schizophrenia,” “cognitive function and schizophrenia,” cognitive function in FEP,” and “cognition in first- episode psychosis,” up to and including published papers June 2013. Criteria for inclusion were FEP, with cognitive measures (data on mean ± SD) for both patients and controls. Twenty-four articles fulfilled these criteria’s. The qualifying studies are presented in Tables 1–5, which include, for each cognitive domain area, the bibliographic reference, the cognitive tests used, and the degree of the impairment identified. Effect sizes were computed using Cohen’s *d* (16). According to Rosenthal and Rosnow (17), effect sizes were considered small for values between 0.20 and 0.50, moderate for values between 0.50 and 0.80, and large for values greater than 0.80. The effect sizes reflect those reported in the original published studies. For papers that did not report the effect size, the effect size was calculated on the basis of the mean and SD in the patient and the control groups.

**Table 1:** **Studies comparing patients with first-episode psychosis and healthy controls on general cognitive ability**.

Cognitive domain meta-analytic study	Study variable	Effect size	
Hill et al. (18)	Wechsler scales IQ	Matched for IQ
Leeson et al. (19)	Wechsler scales IQ	Matched for IQ
Bilder et al. (3)	Wechsler scales (performance IQ)	1.50	
Bilder et al. (3)	Wechsler scales (verbal IQ)	1.53	
Bilder et al. (3)	Wechsler scales IQ (performance and verbal subtests)	1.71	
Chan et al. (20)	Wechsler scales IQ (performance and verbal subtests)	0.29	
Aas et al. (21)	Wechsler scales IQ (performance and verbal subtests)	1.19	
Zanelli et al. (12)	Wechsler scales IQ (performance and verbal subtests)	0.66	All FEP
Zanelli et al. (12)	Wechsler scales IQ (performance and verbal subtests)	1.52	SCH
Zanelli et al. (12)	Wechsler scales IQ (performance and verbal subtests)	0.53	Other psychosis
Leeson et al. (19)	Pre-morbid IQ	0.39	
Joyce et al. (22)	Pre-morbid IQ	0.50	
Mathes et al. (23)	Pre-morbid IQ	1.05	
Zanelli et al. (12)	Pre-morbid IQ	0.54	All FEP
Zanelli et al. (12)	Pre-morbid IQ	0.79	SCH
Zanelli et al. (12)	Pre-morbid IQ	0.45	Other psychosis
Bilder et al. (3)	Pre-morbid IQ	1.00	
Aas et al. (21)	Pre-morbid IQ	1.09	
Hermens et al. (24)	Pre-morbid IQ	0.82	

*ALL FEP = schizophrenia, bipolar disorders, depressive psychosis, psychosis NOS; SCH = schizophrenia*.

### General intellectual function

General cognition is usually assessed in two ways. Firstly, general cognitive ability is measured by a set of multiple cognitive tests such as the Wechsler Adult Intelligence scale (WAIS). The WAIS is based on multiple performance and verbal cognitive tests, which together are believed to reflect general cognitive function. Secondly, general cognition is assessed with a single reading test, such as the National reading test (NART) (3) or the Wechsler Test of Adult Reading test (WTAR). The NART and the WTAR are reading tests which both estimate cognitive ability by reading a list of words, with the IQ level estimated on the basis of mistakes made in the pronunciation of the words listed (38). In the literature on FEP studies, the NART or the WTAR (or equivalent schedules) are normally used to assess pre-morbid intelligence, an estimate of intelligence level achieved before illness onset, while the WAIS is usually used as an estimate of current IQ (12).

### Executive function, attention, and working memory

The term “executive functioning” is often used as a synonym for frontal lobe functioning, and to indicate higher cognitive functioning of the prefrontal cortex (2, 38). Executive function consists of loosely related higher-order cognitive processes, including: problem solving, planning, initiation, hypothesis generation, cognitive flexibility, decision making, regulation, and judgment and working memory (38). Working memory is conceived as a limited capacity storage for retaining information over a short time (1–2 min), and performing mental operations of the content during this period (38). Moreover, the context of the working memory may originate from both sensory inputs as well as from stored long-term memory (38). Attention can be defined as the gateway for information flow to the brain. Attention is a complex system that allows the individual to filter relevant and irrelevant information in the context of internal drives and intentions, hold and manipulate mental processes, and monitor responses to stimuli (38).

### Memory

Memory is a complex process by which an individual registers retains and retrieves information (38). Memory is divided into non-verbal and verbal memory, with verbal memory including declarative memory and non-declarative memory. Declarative memory includes both episodic memory (memory for events) and semantic memory (memory for facts). Declarative memory includes an understanding of something being learned, while non-declarative memory can take place without conscious awareness (38).

Several tests are used to assess memory, and the Wechsler Memory Scale (WMS) Logical memory test has been widely used to assess declarative memory in psychosis. In the logical memory task, the participant is asked to recall a short story, immediately after hearing the story, and again after a delay of 30 min (38). Other widely used verbal memory tasks in psychosis research are the Rey Auditory Verbal Learning Test (RAVLT) and the California Verbal Learning Test (CVLT), assessing memory functioning through the use of a word-list learning paradigm, which includes an immediate and delayed component similar to the Logical memory (38). Non-verbal memory is assessed by tests evaluating the ability to recall a specific visual design, including an immediate and delayed aspect, with Visual Reproduction and the Rey–Osterrieth Complex Figure Test being the most frequent tests used in the literature (38).

### Processing speed

Processing speed refers to the speed at which different cognitive tasks are completed. It normally involves a simple task, which is timed on completion. The importance of processing speed lies in its relevance to many higher cognitive operations, such as perceptual processes, encoding, and retrieval (38).

### Motor speed

Deficits in motor performance are seen in many neurological disturbances. The most frequently used motor tests in FEP are grooved pegboard and finger tapping. The grooved pegboard contains twenty-five holes with randomly positioned slots and pegs which have a key along one side. Pegs must be rotated to match the hole before they can be inserted as quickly as possible. For the finger tapping task the examinee is instructed to tap as rapidly as possible using index finger for a specific amount of time. For both grooved pegboard and finger tapping performance with the preferred hand usually demonstrates best performance (38).

## Results

### General intellectual function

Overall, patients with FEP scored significantly worse on current IQ assessed by the WAIS battery compared to controls (see Table 1). The largest effect size across FEP studies was observed for the WAIS full scale IQ (based on performance and verbal tasks from the WAIS), with an effect size of 1.71 (3). Significant differences between patients and controls have also been replicated in studies using NART or WTAR scores to measure pre-morbid IQ. Thus in our own work (21), we found a significant difference between NART scores in patients and controls, with an effect size of 1.09. Significant differences between patients and controls NART scores are also shown in other studies, with effect sizes varying between 0.39 and 1.05.

### Executive function, attention, and working memory

Patients with FEP show impairment in executive performance, with effect sizes ranging from 0.25 to 1.86, with the most profound deficits observed Mohamed et al. (1) on Wisconsin Card Sorting Test (WCST) perseverative errors, with an effect size of 1.86 (see Table 2). Working memory follows with an effect size up to 1.42, followed by attention (effect size up to 1.30).

**Table 2:** **Studies comparing patients with first-episode psychosis and healthy controls on executive function, attention and working memory**.

Cognitive domain meta-analytic study	Study variable	Effect size
**EXECUTIVE FUNCTION**		
Mohamed et al. (1)	Fluency (total)	0.75
Rodriguez-Sanchez et al. (25)	Fluency (total)	0.86
Ngoma et al. (26)	Fluency (total)	0.76
Zabala et al. (27)	Fluency (total)	0.82
Addington and Addington (28)	Semantic fluency	1.02
Addington et al. (29)	Semantic fluency	1.16
Ma et al. (30)	Semantic fluency (several measures)	0.55–0.58
Chan et al. (20)	Semantic fluency	0.71
Pena et al. (31)	Semantic Fluency	1.49
Addington et al. (29)	Phonemic fluency	0.44
Addington and Addington (28)	Phonemic fluency	0.57
Pena et al. (31)	Phonemic fluency	1.57
Brickman et al. (32)	Trail making test part B	1.10
Ma et al. (30)	Trail making test part B	0.79
Addington and Addington (28)	Trail making test part B	0.97
Addington et al. (29)	Trail making test part B	0.82
Chan et al. (20)	Trail making test part B	0.33
Rodriguez-Sanchez et al. (25)	Trail making test part B	1.07
Ngoma et al. (26)	Trail making test part B	0.80
Aas et al. (21)	Trail making test part B	1.19
Hermens et al. (24)	Trail making test part B	1.10
Perez-Iglesias et al. (33)	Trail making test part B	1.23
Gonzalez-Blanch et al. (34)	Trail making test part B	1.05
Gonzalez-Blanch et al. (35)	Trail making test part B	1.16
Mohamed et al. (1)	Trail making test*	1.41
Zabala et al. (27)	Stroop–color word interference	0.62
Ma et al. (30)	Stroop–color word interference	0.53
Gonzalez-Blanch et al. (35)	Stroop–color word interference	0.57
Mohamed et al. (1)	Stroop–color word interference	0.29
Ngoma et al. (26)	Stroop–color word interference	0.25
Zabala et al. (27)	Stroop (several measures)	0.96–1.28
Pena et al. (31)	Stroop color word test	1.56
Ma et al. (30)	Executive functions (range in reported subgroups)	0.40–0.69
Leeson et al. (19)	Executive function (general)	0.40
Liu et al. (36)	MSET total	1.06
Addington et al. (29)	WCST categories	0.53
Ma et al. (30)	WCST categories	0.61
Addington and Addington (28)	WCST categories	0.64
Chan et al. (20)	WCST categories	0.61
Pena et al. (31)	WCST categories	0.81
Ngoma et al. (26)	WCST categories	0.86
Liu et al. (36)	MWCST categories	0.98
Addington et al. (29)	WCST perseverative errors	0.62
Mohamed et al. (1)	WCST perseverative errors	1.86
Mohamed et al. (1)	WCST perseverative errors	0.75
Pena et al. (31)	WCST perseverative errors	0.51
Addington and Addington (28)	WCST perseverative errors	0.79
Ma et al. (30)	WCST perseverative errors	0.87
Zabala et al. (27)	WCST perseverative errors and errors (several measures)	0.80–0.96
Chan et al. (20)	WCST perseverative errors	0.64
Liu et al. (36)	MWCST perseverative errors	1.11
**WORKING MEMORY**		
Leeson et al. (19)	Spatial span (several tests)	0.42–0.49
Addington et al. (29)	Letter-number span	1.00
Gonzalez-Blanch et al. (35)	Letter-number sequencing test	0.80
Pena et al. (31)	Letter-number sequencing task	1.42
Ngoma et al. (26)	Letter-number sequencing task	1.42
Zabala et al. (27)	Letter-number sequencing task	0.66
Mohamed et al. (1)	Digit span total	0.55
Ma et al. (30)	Digit span total	0.81
Joyce et al. (22)	Spatial working memory CANTAB	1.11
Aas et al. (21)	Spatial working memory (CANTAB) several tests	0.94–1.01
**MAINTENANCE**		
Zabala et al. (27)	Digit span forwards	0.84
**MAINTENANCE + MANIPULATIONS**		
Gonzalez-Blanch et al. (34)	Digit span backwards	1.03
Pena et al. (31)	Digit span backwards	1.11
Zabala et al. (27)	Digits span backwards	0.84
Ma et al. (30)	Arithmetic	0.77
Leeson et al. (19)	Arithmetic	0.22
**ATTENTION**		
Zabala et al. (27)	Continuous performance test	0.44
Zabala et al. (27)	Continuous performance test	0.61
Kleinlogel et al. (37)	Continuous performance test	0.82
Mohamed et al. (1)	Continuous performance test	1.56
Perez-Iglesias et al. (33)	Continuous performance test	0.95
Gonzalez-Blanch et al. (35)	Attention tests (several tests)	0.64–1.42
Rodriguez-Sanchez et al. (25)	Attention index	1.30
Ngoma et al. (26)	Attention index	1.22
Bilder et al. (3)	Attention index	1.15
Addington and Addington (28)	Attention test (Span)	0.07
Addington et al. (29)	Attention tests (several)	0.29–0.39
Chan et al. (20)	Attention test	0.48
Joyce et al. (22)	Attention set shifting CANTAB	0.52

*WCST, Wisconsin card sorting test; MSET, modified six elements test; MWCST, modified Wisconsin card sorting test; trail making test time, not specified whether this is Trail A or Trail B*.

### Memory

Several studies in FEP have shown significant impairments in memory, particularly in verbal declarative episodic memory (see Table 3). The effect sizes of verbal episodic memory deficits vary from 0.26 to 2.10, and the effect sizes for non-verbal memory vary from 0.38 to 1.65. As shown by the high effect size reported from studies on FEP, memory impairment is the most, or one of the most, impaired cognitive domains already at illness onset.

**Table 3:** **Studies comparing patients with first-episode psychosis and healthy controls on memory function**.

Cognitive domain meta-analytic study	Study variable	Effect size
Hermens et al. (24)	Immediate verbal memory (general)	1.81
Zabala et al. (27)	Immediate verbal memory (general)	1.62
Gonzalez-Blanch et al. (35)	Immediate verbal memory (general)	0.81
Addington and Addington (28)	Immediate verbal memory (general)	1.13
Ma et al. (30)	Immediate verbal memory (general)	0.73
Hill et al. (18)	Immediate verbal memory (general)	0.58
Chan et al. (20)	Immediate verbal memory (general)	0.45
Aas et al. (21)	Immediate verbal memory (general)	1.85
Ma et al. (30)	Immediate verbal memory (general)	0.46
Addington et al. (29)	Immediate verbal memory (general)	2.10
Mohamed et al. (1)	Immediate verbal memory (general)	1.34
Rodriguez-Sanchez et al. (25)	Verbal memory (both immediate and delayed)	0.80
Bilder et al. (3)	Verbal memory (both immediate and delayed)	1.63
Brickman et al. (32)	Learning	1.79
Hermens et al. (24)	Learning	1.34
Leeson et al. (19)	Learning	0.61
Ngoma et al. (26)	Learning	1.14
Addington et al. (29)	Learning	1.63
Addington and Addington (28)	Learning	0.26
Zabala et al. (27)	Learning	1.47
Mohamed et al. (1)	Delayed verbal memory (general)	1.28
Mohamed et al. (1)	Delayed verbal memory (general)	1.22
Addington et al. (29)	Delayed verbal memory (general)	1.49
Addington et al. (29)	Delayed verbal memory (general)	1.55
Hill et al. (18)	Delayed verbal memory (general)	0.55
Addington and Addington (28)	Delayed verbal memory (general)	0.99
Addington and Addington (28)	Delayed verbal memory (general)	1.26
Ma et al. (30)	Delayed verbal memory (general)	0.82
Chan et al. (20)	Delayed verbal memory (general)	0.64
Gonzalez-Blanch et al. (35)	Delayed verbal memory (general)	0.72
Aas et al. (21)	Delayed verbal memory (general)	1.90
Pena et al. (31)	Delayed verbal memory (general)	1.44
Hermens et al. (24)	Delayed verbal memory (general)	1.50
Perez-Iglesias et al. (33)	Delayed verbal memory (general)	0.75
Zabala et al. (27)	Delayed verbal memory (general)	1.54
**MEMORY–NON-VERBAL**		
Mohamed et al. (1)	Immediate non-verbal memory	0.85
Aas et al. (21)	Immediate non-verbal memory	0.68
Addington et al. (29)	Immediate non-verbal memory	0.38
Addington and Addington (28)	Immediate non-verbal memory	0.86
Bilder et al. (3)	Non-verbal memory index	1.65
Aas et al. (21)	Delayed non-verbal memory	0.39
Ma et al. (30)	Delayed non-verbal memory	0.48
Mohamed et al. (1)	Delayed non-verbal memory	0.92
**RECOGNITION**		
Mohamed et al. (1)	Recognition	1.70
Hill et al. (18)	Recognition	0.58
Aas et al. (21)	Recognition	1.56

### Processing speed

Studies of patients with FEP have shown reduced processing speed in these patients when compared to controls, with effect sizes ranging from 0.33 to 1.69 (see Table 4).

**Table 4:** **Studies comparing patients with first-episode psychosis and healthy controls on processing speed**.

Cognitive domain meta-analytic study	Study variable	Effect size
**PROCESSING SPEED**		
Addington and Addington (28)	Trail making test part A	1.14
Ma et al. (30)	Trail making test part A	0.76
Brickman et al. (32)	Trail making test part A	0.95
Addington et al. (29)	Trail making test part A	0.96
Chan et al. (20)	Trail making test part A	0.45
Aas et al. (21)	Trail making test part A	1.11
Gonzalez-Blanch et al. (34)	Trail making test part A	0.70
Pena et al. (31)	Trail making test part A	1.33
Zabala et al. (27)	Trail making test part A	0.77
Hermens et al. (24)	Trail making test part A	1.01
Ngoma et al. (26)	Trail making test part A	0.33
Gonzalez-Blanch et al. (35)	Trail making test part A	1.07
Ma et al. (30)	Digit symbol coding	1.45
Gonzalez-Blanch et al. (34)	Digit symbol coding	1.69
Mohamed et al. (1)	Digit symbol coding	1.69

### Motor speed

Patients with FEP show impairments on motor speed (with effect sizes varying from 0.36 to 1.26; see Table 5).

**Table 5:** **Studies comparing patients with first-episode psychosis and healthy controls on motor speed**.

Cognitive domain meta-analytic study	Study variable	Effect size
**MOTOR SKILLS**		
Addington and Addington (28)	Pegboard (dominant)	0.72
Addington et al. (29)	Pegboard (dominant)	0.76
Perez-Iglesias et al. (33)	Pegboard (dominant)	1.01
Addington et al. (29)	Pegboard (non-dominant)	0.90
Addington and Addington (28)	Pegboard (non-dominant).	0.79
Rodriguez-Sanchez et al. (25)	Finger tapping left and right hand	0.89
Ngoma et al. (26)	Finger oscillation task	1.26
Brickman et al. (32)	Finger tapping test, left hand	0.36
Mohamed et al. (1)	Finger tapping test, left hand	0.62
Brickman et al. (32)	Finger tapping test, right hand	0.51
Mohamed et al. (1)	Finger tapping test, right hand	0.79

## Discussion

Reviewing the literature on cognitive function in FEP, we found significant impairment (Cohen’s *d* effect size above 0.8 (17) in all cognitive domains investigated; see Tables 1–5. The largest effect size was observed for verbal memory (Cohen’s *d* effect size = 2.10), followed by executive function (effect size = 1.86), and general IQ (effect size = 1.71). Our systematic review focused on FEP and included studies published up to 2013, thus covering studies not included previously in the review by Bozikas and Andreou (13) (included studies up to January 2010); or Lewandowski et al. (14) (included studies up to 31st of March 2010). Moreover, the study by Bozikas and Andreou (13) included only articles with longitudinal data, excluding studies presenting FEP only. Also the study by Lewandowski et al. (14) included articles on cognitive function in chronic schizophrenia and other psychosis. Our study therefore complements these articles, focusing in-depth on the first-episode only. We have also calculated effect sizes based on Cohen’s *d*, providing information about the severity of the impairment, which is not included in the previous reviews described above. Similar to the study of Bozikas and Andreou (13) and Lewandowski et al. (14) we found that already at illness onset patients with FEP are characterized by cognitive impairments across domains. Moreover, our results support different cognitive pattern for schizophrenia and affective psychosis (schizophrenia patients showing the greatest effect size differences in cognitive performance compared to controls). Further, we found more variation in the effect sizes comparing patients and controls on cognitive tests than the study by Reichenberg et al. (2) in chronic schizophrenia, indicating a further consolidation of cognitive impairment over time may be present. These results are similar to Bozikas and Andreou (13) and Lewandowski et al. (14) showing the main reduction in cognition around illness onset, or pre-morbid period, with a relatively stable course, which may become even more pronounced after some years.

Our findings in FEP demonstrate that already at illness onset patients have reduced *IQ scores* compared to controls. Both current IQ and estimated pre-morbid IQ were significantly lower than those of controls, with the greatest impairment in current IQ scores. Indeed, the discrepancy between current IQ and pre-morbid scores is believed to reflect the deterioration in current IQ at or around illness onset. The study by Zanelli et al. (12) show that when we examine schizophrenia versus other psychosis (psychosis NOS and affective psychosis), patients with schizophrenia have larger effect size differences in current IQ, as well as pre-morbid IQ (schizophrenia current IQ effect size = 1.52; pre-morbid IQ effect size = 0.79), compared to other psychosis (current IQ effect size = 0.53; pre-morbid IQ effect size = 0.45). This indicates worse cognitive function in the schizophrenia group both at illness onset and prior to illness onset, compared to patients with other psychosis. Patients in the schizophrenia group also present the greatest deterioration (measured by effect size) comparing current IQ to pre-morbid IQ [schizophrenia effect size current IQ (1.52) minus effect size pre-morbid IQ (0.79) = 0.73, compared to other psychosis: effect size current IQ (0.53) minus effect size pre-morbid IQ (0.45) = 0.08]. A worse cognitive profile in patients with schizophrenia was also found in the study by Hill et al. (39) showing that patients with FEP who developed schizophrenia demonstrated more impaired cognitive performance across domains, compared to patients with affective psychosis. Similar findings are also observed in chronic psychosis (14).

Several studies conducted in FEP have matched patients and controls on general cognition, in order to investigate specific deficits believed to be particularly marked in psychosis. It should be mentioned that matching subgroups on IQ aiming to measure specific cognitive areas believed to be impaired in psychosis may be challenging due to that such a comparison would be contaminated by regression toward the mean (Lord’s Paradox) (40).

### Executive function, attention, and working memory

Although a strong correlation has been found between general intellectual function and other specific cognitive subareas, such as memory and executive functioning, research has also shown impairment of executive functions, such as planning, flexibility and judgment, without any major change in general intellectual status (38). Is the impairment in executive and working memory observed in patients with psychosis a consequence of general cognitive decline, or is executive impairment in psychosis independent from overall cognitive performance? Chan et al. (20) aimed to answer this question in a study of 78 patients with first-episode medication-naïve schizophrenia and 60 controls; they showed that patients with first-episode medication-naïve schizophrenia exhibit specific types of executive dysfunction independently of general cognitive decline. However, Table 2 shows that there is a great discrepancy across studies in terms of effect sizes observed; for example the effect size 0.22 was found for “Arithmetic” in the study by Leeson et al. (19), compared to an effect size of 0.77 in the study by Ma et al. (30). One difference between these two studies was that the study by Leeson et al. (19) had matched the patients and controls for general IQ, while the study by Ma et al. (30) had not. It is therefore important to have in mind whether general IQ has been taken into account in the analysis when interpreting effect sizes for different cognitive subtests.

### Memory

It has been suggested that episodic memory tests could be used to elicit diagnostic differences in a psychosis sample. For example, in the study by Fitzgerald et al. (41) consisting of 83 patients with FEP, decreased performance on verbal memory was reported in patients with schizophrenia compared to patients with affective psychosis, implying diagnostic differences in performance on this specific memory task. Prospective studies also show worse cognitive profile in patients with FEP who later develop schizophrenia across cognitive domains, compared to patients with affective psychosis (12, 39).

### Processing speed

The study by Leeson et al. (19), comparing FEP and healthy controls (matched for general cognition) reported that processing speed was the cognitive domain most impaired in FEP. Leeson et al. (19) also found a relationship between processing speed performance and performance on executive and memory tasks, two known impaired cognitive domains in psychosis, as discussed earlier. This indicates the importance of measuring processing speed when studying patients with psychosis. As processing speed is important for the performance of higher cognitive operations, such as executive functions and memory, it has been suggested that impaired processing speed underlies the abnormalities in these areas (42). On the other hand, to complicate this further, a reduction of processing speed has also been found associated with antipsychotic medication (43).

Below are hypothesis about putative mechanism(s) of the cognitive reduction in FEP, investigating the role of early stress, inflammation and genetic vulnerability.

### Stress and cognition in psychosis

Some previous studies in chronic patients with psychosis, and in healthy controls, report a negative correlation between childhood trauma and cognitive function, particularly decreased scores on general cognitive abilities, memory, and executive function (44–48). This is interesting as similar cognitive areas are found to be most reduced in FEP, comparing patients to controls performance (see Result). The deleterious effect of stress and glucocorticoid on the brain (particularly on hippocampus) is well known in the literature (49, 50), and indeed disorders characterized by increased stress exposure such as depression, post-traumatic stress disorder, chronic fatigue disorder, show cognitive impairment, particularly in memory and in executive function (51–53). Moreover, psychosis is a disorder characterized by high level of stressful events (54), including recent stressful events (55), the inability to cope with life events (56, 57), and childhood trauma (58, 59). It is therefore possible that some of the cognitive impairment in FEP is due to an increased exposure, or vulnerability toward the negative effect of particularly childhood trauma. We will now review the literature supporting this putative mechanism.

### Cortisol level and cognitive function and psychosis

Over two decades have past since the first study investigating Hypothalamic-Pituitary-Adrenal (HPA) axis and cognitive abnormalities in schizophrenia were published (60) and suggested a link between hypercortisolism and cognitive impairment. Since then, several studies have investigated HPA axis and cognitive function in long-term chronic schizophrenic patients. The first study by Saffer et al. (60) consisted of 11 patients with type II schizophrenia (mainly negative symptoms), 34 controls, 30 patients with schizophrenia with type 1 (mainly positive symptoms), and 9 patients with mixed type I and type II schizophrenia (both negative and positive symptoms). This study found a strong negative correlation between dexamethasone abnormalities (dexamethasone non-suppressors) and cognitive performance in type II schizophrenics, implying a relationship between HPA axis hyperactivity and cognitive impairment. Furthermore, Walder et al. (61), evaluating 18 patients with chronic psychosis, 7 patients without psychosis, and 19 healthy controls, found that, in the entire sample, cortisol levels were negatively correlated with performance on memory and executive tasks.

Also in FEP an abnormal HPA axis has been demonstrated (55) showing higher cortisol levels during the day in the patients compared to the controls. An interesting closed-loop model, first hypothesized by Lupien (62), gives a possible explanation for the relationship between cortisol level and cognition in schizophrenia. The closed-loop model works in the following manner: basal cortisol level increases symptoms severity, which again impacts on the encoding of incoming information into long-term memory store; this leads to difficulties discriminating between relevant (threat) and irrelevant (non-threat) information, which again further increases the reactivity to stress.

In our recent paper on patients with FEP (21), we found that patients performed significantly worse on all cognitive domains compared to controls. In patients only, a blunted cortisol awakening response (that is, more abnormal) was associated with a more severe deficit in verbal memory and processing speed, supporting a role for the HPA axis, as measured by cortisol awakening response, in modulating cognitive function in patients with psychosis.

### Childhood trauma and cognitive function in psychosis

Six studies in the literature have investigated cognition and early trauma in people with psychosis, four in individuals including chronic illness phase. The first study by Lysaker et al. (45) was conducted in males with schizophrenia spectrum disorder, and no comparison control group was used. The authors found that male patients with childhood trauma (sexual abuse) had impaired processing speed, working memory, and executive function compared with patients without abuse (45). The second study by Schenkel et al. (47) included 15 female and 25 males with schizophrenia spectrum disorder, with no comparison control group. The authors found that patients with schizophrenia or schizoaffective disorder with a history of childhood trauma showed a decreased score on learning and visual context processing compared to non-abused patients. Similar findings were found in the study by Shannon et al. (48) in a sample of 85 patients (67 males and 18 females) with chronic schizophrenia; here patients with childhood trauma scored significantly lower on working memory as well as verbal memory tasks, even after controlling for pre-morbid IQ and depressive symptoms. Similar findings were observed in our own examination of the large AESOP study of FEP which comprised of 138 patients (73 males and 65 females) and 138 controls (64 males and 74 females), showing an association between childhood trauma and reduced performance on attention, concentration and mental speed, language, and verbal intelligence; however this was mainly driven by male patients with affective psychosis (44), whilst in controls only one subtest (performance intelligence) was reduced in the group with high exposure of childhood trauma, and in males only. Also in an independent study including 406 patients with a mean duration of illness of 3 years showed a strong negative correlation between cognitive function and a history of childhood trauma; here childhood trauma was associated with a reduction in cognitive function across cognitive domains in patients with schizophrenia spectrum- and bipolar disorders, in particular working memory and executive function, as well as general cognition. Moreover, these dysfunctions were driven by underlying deficits in general cognitive tasks as measured by the Wechsler Abbreviated Scale of Intelligence (WASI) (63). Only two studies have found negative, or inconclusive, findings between cognitive function and early trauma in FEP (21, 64), with the latter and largest study showing an effect of childhood trauma on cognitive function in controls, but not in patients (64).

### Potential biological mechanisms

Since patients with psychotic disorders – in addition to being more exposed to trauma – also demonstrate abnormalities in the HPA axis (55), immunological disturbances (65, 66), together with cognitive and brain structure abnormalities (6, 12, 21, 67), it is of interest to investigate the link between stress exposure and biological stress parameters to cognitive abnormalities in psychosis. Based on the findings outlined above, it is particularly intriguing to investigate to what extent patients with psychotic disorders also have a genetic vulnerability toward abnormal stress response that may add to – or interact with the effects of early trauma, and cognitive impairments.

There are several indications of an abnormal HPA axis in patients with psychotic disorders irrespective of early trauma. This includes findings of an increased pituitary volume in patients with FEP compared to controls (68, 69). There are also signs of an altered or increased systemic cortisol metabolism (70–72) with links to genetic markers in psychosis (71), which might interact with the effect of stressful life event. The idea of a genetic predisposition to an abnormal stress response is supported by indications that not only patients with psychosis have abnormal stress responses, but also their relatives (73). Moreover, increased cortisol levels correlate with smaller hippocampal volume in psychosis (74). There is also an interaction between glucocorticoids and serotonin in the central nervous system (CNS) as glucocorticoids regulate both tryptophan hydroxylase and the expression of several serotonin receptors (75). In addition, the functional polymorphism in the promoter region of the serotonin transporter gene (5-HTTLPR) has been linked to altered stress response, as carriers of the short (s-) allele have increased negative psychological reactions and stress hormone release compared with carriers of the long (l-) allele (76). Indeed, our study Aas et al. (76) demonstrated that patients with an early psychotic illness who were carriers of the short (s-) allele of the serotonin transporter gene (5-HTTLPR), exposed to high levels of childhood trauma (physical neglect and abuse) had significantly poorer cognitive functioning, than all other groups. Patients with psychosis also show reduced brain-derived neurotropic factor (BDNF) levels both in the brain (77), and in serum and plasma (77–79), which may be related to their cognitive performance (80). The BDNF gene has at least one functional variant, the SNP (rs 6265) resulting in a Valine (val) to Methionine (met) substitution at codon 66 of the proBDNF. The low active met allele is here related to reductions in BDNF release (81). Recent research in FEP have found associations between reduced BDNF gene expression levels, childhood trauma, increased inflammation, and smaller hippocampal volume (82). Reduced BDNF levels are also observed in bipolar patients exposed to childhood trauma (83), as well as linked to cognition in animals (84). It is well known that patients with psychosis show brain structural abnormalities compared to healthy controls (67, 85, 86). A recent meta-analyses in healthy individuals indicate hippocampus volume reductions in met carriers compared to homozygotic val carriers (87); this is an area of the brain important for memory. In psychosis, met carriers usually demonstrate larger ventricles, more CSF, and reduced frontal gray matter volume (88, 89), even if there are inconsistent results (90). While the study by Mondelli et al. (82) did not genotype, their study support the role of BDNF on brain structures, as well as a relationship between childhood trauma and BDNF levels. Childhood trauma may thus represent a significant factor influencing cognitive function in psychosis, mediated through an effect on BDNF. Indeed our recent study in patients with a psychotic illness (80) support this hypothesis, showing that BDNF val66met-met carriers reporting high levels of childhood trauma demonstrate significantly reduced executive function/working memory as well as smaller hippocampal volume, compared to all other groups.

Several lines of evidence have implicated the immune system in the development of severe psychiatric disorders, and increased inflammation is found in depression (91–93), as well as in psychosis (65, 66, 94). Recent GWAS studies clearly indicate immune genes as susceptibility genes for schizophrenia (92, 95). Increased inflammation is also found to be associated with cognitive impairments in animals (96). Pilot data from a small sample of patients with FEP and healthy controls show a relationship between increased inflammation parameters (IL-8 and IL-6) and cognitive impairments across both groups (97), indicating that immune markers may be related to cognitive function in psychosis. More studies should investigate this further, aiming to understand the cognitive reduction seen in FEP compared to healthy controls.

### Epigenetic research related to schizophrenia risk and cognitive function

Recent epigenetic research related to schizophrenia risk, links early stress (hypoxia and perinatal stress) to methylation and altered gene expression associated with behavior and cognitive changes (98). Perinatal stress has also been found to be related to reduction of Brain-derived neurotrophic factor (BDNF) in the brain of adult rats (84). Riva et al. (84) also show that perinatal stress in male rats is related to reduced performance on the object recognition test in the adult rat. Riva linked the above to epigenetic changes, explaining that perinatal stress increases methylation, which again is related to a reduced transcript of BDNF, and cognitive changes. Riva et al. (84) has also demonstrated that perinatal stress is associated with reduced upregulation of BDNF on the forced swim test in adult rats, linking this to impaired ability to cope under stressful situations in rats exposed to perinatal stress. Finally, Riva et al. (84) also found that epigenetic changes in response to stress are seen across genes: methylome analysis in the prefrontal cortex of rats exposed to perinatal stress showed that a large number of genes (3,386 genes) were methylated differently from controls. These included genes linked to bipolar disorders and schizophrenia, such as CACNA1C and DISC1, as well as the COMT gene. Further research should therefore investigate links between early stress, and epigenetic changes of relevant genes across genome, aiming to understand mechanisms behind cognitive abnormalities in patients with FEP.

### Limitations

This study has several limitations which should be acknowledged: we decided to focus on only some aspects which may influence cognitive function (i.e., stress, and interactions between stress and genetic factors, as well as inflammation), and we did not have the possibility to go into depth on other important aspects, such as time of illness onset (early illness related to worse cognitive function), possible gender differences (males lower performance), the influence of antipsychotics and medication, and the duration of treatment. Although all these patients are FEP, the exact level of treatment varied between the studies (see Supplementary Material for an overview of recruitment in the different papers discussed). Moreover other environmental factors such as cannabis have both been associated with increased risk of the illness, as well as influencing cognitive function, however due to space and focus of the paper, we focused on the role of stress and inflammation and genetics related to these factors as possible explanation model. Only the Cohen’s *d* and not the confidence intervals for the Cohen’s *d* are included in this study. However, we have included in the Supplementary Material a detailed overview of the studies included aiming to facilitate interpretations of the findings. As already mentioned matching subgroups on IQ aiming to measure specific cognitive areas believed to be impaired in psychosis may also be challenging due to that such a comparison may be influenced by regression toward the mean (Lord’s Paradox) (40).

### Summary

We have demonstrated that cognitive impairment across domains (up to and above severe level based on Cohen’s *d* effect size) is present already at illness onset, as shown in FEP studies. However, differences in levels of impairment are observed between studies, as well as within domains, indicating that compared to chronic schizophrenia (2), a further consolidation of cognitive impairment over time may be present.

**Figure 1:**
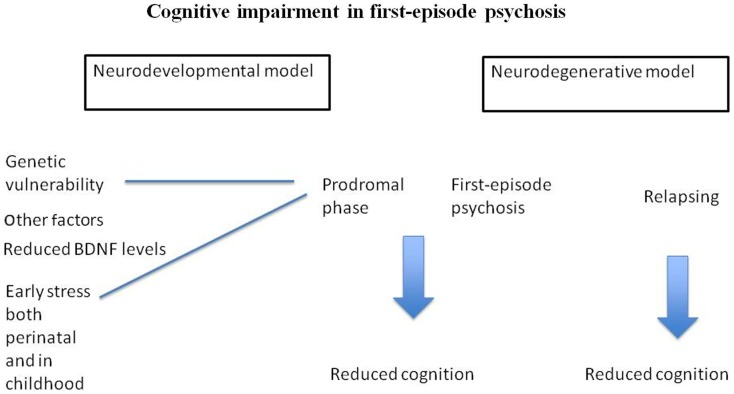
**Suggests that cognitive impairment in FEP is related to neurodevelopmental abnormalities**. Figure 1 also postulate that stress, as well as genetic vulnerability may be part of the complex etiology behind cognitive impairment in psychosis.

The research into trauma, stress, and HPA axis disturbances (including gene interactions) is one of the areas where we have some knowledge about how specific environment and genetic factors influence cognition (Figure 1). Indeed, early stress may have long-lasting changes on cognitive function by affecting expression of relevant genes. This is the start of a new line of research aimed at further understanding the complex etiology behind cognitive abnormalities in psychosis. Based on published biological studies, we propose that some of these impairments may be due to these subjects’ increased sensitivity to the negative effect of stress, and genetic vulnerability, affecting the HPA axis, the immune function, and neuroplasticity. More studies are needed aiming to understand the complex etiology of cognitive impairments in psychosis.

## Conflict of Interest Statement

The authors declare that the research was conducted in the absence of any commercial or financial relationships that could be construed as a potential conflict of interest.

## Supplementary Material

The Supplementary Material for this article can be found online at http://www.frontiersin.org/Journal/10.3389/fpsyt.2013.00182/abstract
