# Supplementary material for: A Systematic Review of Cognitive Function in First-Episode Psychosis, Including a Discussion on Childhood Trauma, Stress, and Inflammation
Source: Front Psychiatry. 2014 Jan 8;4:182. doi: 10.3389/fpsyt.2013.00182 (PMC3884147; doi:10.3389/fpsyt.2013.00182)
Supplement: Supplementary file 1 [file 65931_Aas_DataSheet1.PDF]

SUPPLEMENTARY MATERIAL
Demographic overview of the articles included in the first-episode psychosis and cognitive function review

	**Age group (mean±SD) patients controls**	**Gender m/f patients controls**	**Diagnostic criteria**	**Matched /controlled for IQ or education**	**Number of subjects patients controls**	**Selection of subjects**	**Medication**
(Aas *et al.* 2011b)	30.1±7.2<br>27.5±4.8	20/10<br>18/8	27 were classified with schizophrenia or delusional disorder, and 3 were classified as "other psychosis"	----	30<br>26	Both in and outpatients recruited within 6months of first contact with mental health services for psychosis. Controls were recruited from the same catchment area as the patients.	5 drug naive, 22 were taking atypical, and 3 were taking typical antipsychotic medication.
(Aas *et al.* 2011a)	30.6±10.9<br>32.2±9.3	73/65<br>64/74	83 were classified as schizophrenia, 55 were classified with affective psychosis (29 mania, and 26 were classified with depression).	----	138<br>138	Both in and outpatients recruited within the first year of contact with mental health services for psychosis. Controls were recruited from the same catchment area as the patients.	Medication details available for 79 patients: 10 were drug naive, 19 were on atypical medication, 14 on typical antipsychotics and 16 were using both atypical and typical medication.
(Addington *et al.* 2003)	24.7± 8.19<br>22.7±7.4	204/108<br>38/28	124 were classified with schizophrenia,	----	312<br>66	Admitted to the Calgary Early Psychosis Program	11% were drug naive; 89.8% were taking atypical

			120 as schizophreniform, 5 with delusional disorder, 12 with brief psychotic disorder, 44 Psychotic disorder NOS, 7 drug induced psychosis.			(EPP), all had a first episode of psychosis with a maximum of 3 months treatment.	medication.
(Addington and Addington, 2008)	25.1± 8.09<br>21.7±6.05	30/20<br>33/22	64% were classified with schizophrenia, 24% as schizophreniform, 2% delusional disorder, 2% brief psychotic disorder, 6% psychotic disorder NOS, and 2% schizoaffective disorder.	----	50<br>55	Admitted to the Calgary Early Psychosis Program (EPP), all had a first episode of psychosis with a maximum of 3 months treatment.	87.5% were taking atypical medication.
(Bilder *et al.* 2000)	25.7±6.3<br>25.3±6.5	55/39<br>24/12	70 schizophrenia spectrum; 24 affective schizophrenia.	-----	94<br>36	Inpatients admitted with first episode psychosis with less than 12 weeks of antipsychotic treatment. Controls were recruited by the local newspaper and within the	All patients had less than 12 weeks of antipsychotic treatment.

						medical centre, and selected based on similar age and gender as the patients.	
(Brickman *et al.* 2004)	16.1 ±2.0<br>16.9±2.4	15/14<br>9/8	18 schizophrenia, 4 schizoaffective disorders, 5 bipolar disorder, 1 major depressive with psychosis, 1 psychotic disorder NOS (the latter dropped out of the study).	-----	29<br>17	Recruited at their first psychotic experience at community physicians and emergency department clinicians. Controls were recruited through local area newspaper advertisement and word of mouth.	All patients were drug naive.
(Chan *et al.* 2006)	28.5 ±9.8<br>27.9±9.1	49/29<br>19/41	All had first episode schizophrenia.	both	78<br>60	Recruited as part of the Assessment service for Young People with Psychosis in queen Mary hospital.	All patients were drug naive.

(Gonzalez-Blanch *et al.* 2011)	26.8±7.3<br>25.9±6.3	85/46<br>13/15	77 schizophrenia, 34 schizophreniform, 8 brief psychotic disorder, 7 psychosis NOS, 4 schizoaffective disorder, 1 schizotypical personality disorder	-------	131<br>28	Recruited as part of the Cantabria Intervention programme of First-Episode Psychosis at the university hospital (Santander, Spain).<br>Controls were recruited from the local area via advertisement.	88 (67%) used atypical antipsychotic medication.<br>43 (32.8%) used typical antipsychotic medication.
(Gonzalez-Blanch *et al.* 2006)	26.3±7.6<br>26.5±6.0	26/16<br>26/17	60% schizophrenia, 5% schizoaffective disorder, 21% schizophreniform, 14% psychosis NOS	No difference in education level between patients and controls	42<br>43	Recruited as part of the<br>Cantabria intervention program of first-episode psychosis (PAFIP).<br>The PAFIP was designed to provide a comprehensive and multidisciplinary mental health care serving a population of 555.000 in<br>the catchment area of Cantabria, Spain	Baseline cognitive assessment was carried out as soon as possible during the 72 h after the pharmacological treatment was initiated.
(Hermens *et al.* 2010)	22.5±3.2<br>22.6±2.8	12/5<br>7/9	1 schizophrenia, 3 schizoaffective	------	17<br>17	Recruited to a specialised tertiary	15 patients were treated with atypical

			disorder, 5 schizophreniform, 2 bipolar with psychotic features, 6 major depressive disorder with psychotic features			referral service for assessment and early intervention of mental health problems in young people. Controls were recruited from the same catchment area.	antipsychotics, 9 were taking antidepressants and 3 were taking mood stabilizers.
(Joyce *et al.* 2002)	25.7±8.0<br>26.1±5.2	107/29<br>49/32	133 schizophrenia, 3 schizophreniform disorder	Controlled for IQ	136<br>81	As soon as possible after presentation to mental health population with first-episode psychosis. Controls were recruited rom the same catchment area.	No more than 12 weeks of antipsychotic treatment. 59 used atypical antipsychotic medication, and 64 typical antipsychotic medication.
(Hill *et al.* 2004)	26.3±8.9<br>28.0±9.9	58.1% males<br>55.2% males	54 schizophrenia, 9 schizoaffective	Matched for IQ	62<br>67	Recruited first episode psychosis to the university of Pittsburgh medical centre. Controls recruited from the community.	All patients were drug naive.
(Kleinlogel *et al.* 2007)	25.0±4.9<br>24.6±4.3	15/3<br>15/3	schizophrenia	-----	18<br>18	------	9 were drug naive, the rest were on atypical or typical antipsychotic medication.
(Leeson *et al.*	26.8 ±7.8	34/19	schizophrenia,	Individually	53	The patients were	Less than 12 weeks

2010)	26.5±6.70	34/19	schizophreniform, or schizoaffective disorder	Matched for IQ (WAIS IQ within 3 points)	53	recruited for this study as part of a longitudinal study of first-episode psychosis inWest London. Healthy volunteers served as control subjects and were recruited from the same catchment area as patients by advertising in local colleges and hospitals. For the controls, participants were excluded if they had a history of psychiatric illness in themselves or their first-degree relatives.	of antipsychotic treatment. At the time of baseline testing, 3 patients were not being prescribed antipsychotic medication, 2 were being prescribed first-generation antipsychotics, and 48 second-generation antipsychotics. Two patients were also being prescribed anticholinergic medication.
Liu 2011	27.6±1.0<br>26.8±10.3	21/10<br>14/17	29 Schizophrenia<br>4 schizoaffective	---	31<br>31	Part of a large cohort of first episode schizophrenia in china. Controls were recruited from the general population.	Medication naive.

Mathes 2005	22.0±3.2<br>25.0±8.0	40/15<br>40/16	55<br>schizophreniform	Controlled for premorbid IQ	55<br>56	Both patients and controls were selected from a larger database comprising subjects from several ongoing projects. Controls were from the same catchment area as the patients and recruited through advertisements.	33 had received atypical medication, 14 had received a low dose of typical antipsychotic medication. 6 were also treated with anticholinergic medications. 2 were neuroleptic naive at time of neurospycholological assessments; 4 did not have data on medication.
Ma 2007	25.4±7.0<br>39.7±15.6	104/103<br>51/82	First-episode schizophrenia	Regressed on years of education	207<br>133	Both in and outpatients clinics.	163 were neuroleptic naive, 44 had taken antipsychotic medication at a low dose with less than three days duration prior to cognitive testing.
Mohammed 1999	26.1±8.1<br>25.5±5.7	53/41<br>162/143	First-episode psychosis.	Adjusted for education	94<br>305	Recruited at the general psychiatric ward and mental health clinical research centre, University of Iowa hospital and clinics. Controls were recruited from the	73 were drug naive, 14 received treatment for less than one week, and the remaining 7 had received treatment for less than 2 weeks.

						community trough newspapers advertisements.	
Rodrigques-sanches 2007	26.9±7.3<br>25.7±6.3	81/45<br>13/15	77 schizophrenia, 34 schizophreniform disorder, 8 brief psychotic disorder, 7 psychosis NOS	Controlled for vocabulary test wais – III (as an estimate of premorbid IQ). As well as years of education.	126<br>28	From a large epidemiological and longitudinal intervention program of first-episode psychosis, Spain. Both in and out patients clinics. Controls recruited from the local area.	42 haloperidol,<br>41 Olanzapine, 43, Risperidone
Ngoma 2010	28.0±7.1<br>28.1±7.1	102/86<br>66/87	50 schizophreniform disorder, 70 schizophrenia, 68 brief psychotic episode.	Controlled for education.	188<br>153	All patients apart from one were patients with first episode psychosis recruited at the Centre Neuro-Psychopathologique (CNPP) and the centre de santé mentale telema of Kinshasa, Kongo.	154 typical antipsychotics, 4 with mixed typical and atypical antipsychotic medication.
Pena 2011	28.5±7.5<br>controls: Do not say, apart from that do not differ on age compared to patients	68.8% of the patients were male.<br>Controls: Do not say, apart from that do not differ on	5.8% schizophrenia, 30.2 % schizophreniform disorder, 32.6% brief psychotic disorder, 22.1%	-----	86<br>34	Patients recruited at the Early Psychosis Program from the first-episode Psychosis Unit of Cruces Hospital (Spain). 34 controls	Antipsychotic medication was administered.

		gender compared to patients	bipolar disorder, 4.7% delusional disorder, 2.3% drug induced psychosis, 2.3.% major depressive disorder with psychotic features			screened for past or current psychiatric illness, sensory deficits or medical conditions related to the central nervous system.	
Perez-inglesias 2010	Patients and controls matched on age	Patients and controls matched on gender	All with a broad first episode of schizophrenia spectrum diagnosis	Patients and controls matched on years of education	49<br>41	Patients recruited as part of the large first episode psychosis study program of Cantabria. Spain.	All patients agreeing to participate in the study were randomly assigned to either aripiprazole, quetiapine, or ziprasidone.
Zabala 2010	15.5±1.8<br>15.2±1.9	71/36<br>62/36	36 schizophrenia, 19 bipolar, 52 other psychosis.	----	107<br>98	Part of the CAFEPS. Patients were recruited from child and adolescent psychiatry units at six university hospitals. The six hospitals were located in Madrid, Barcelona, Vitoria, Santander, and Pamplona.	96.2% of the patients received an atypical antipsychotic treatment at time of assessment, time 5±9.6 weeks. 2 received typical antipsychotic medication.
Zanelli 2010	30.2±9.7<br>37.2±12.9	101/86<br>77/100	65 schizophrenia 46 other psychosis, 39	Both with and without controlling	187<br>177	Part of the AESOP, first-episode psychosis study,	No information on medication

			depressive psychosis, 37 bipolar or mania,	for IQ (follow-up analysis controlling for IQ. (Only the unadjusted scores are included in this review)		and recruited form south-east London, Nottingham and Bristol. Also see article: Morgan 2006.	
